# Intraoperative hemodynamic management in abdominal aortic surgery guided by the Hypotension Prediction Index: the Hemas multicentric observational study

**DOI:** 10.1186/s44158-024-00222-x

**Published:** 2025-02-13

**Authors:** Enrico Giustiniano, Fulvio Nisi, Federica Ferrod, Giulia Lionetti, Cristina Viscido, Antonio Reda, Federico Piccioni, Gabriella Buono, Maurizio Cecconi

**Affiliations:** 1 Department of Anesthesia and Intensive Care, IRCCS Humanitas Research Hospital, Milan, Italy; 2 Department of Cardiovascular Anesthesia and Intensive Care Unit, AO Mauriziano Umberto I, Turin, Italy; 3 Department of Biomedical Sciences, Humanitas University, Milan, Italy

**Keywords:** Abdominal aorta, Hemodynamic monitoring, Vascular surgery, Intraoperative hypo-tension, Open aortic repair

## Abstract

**Background:**

Intraoperative hypotension (IOH) during non-cardiac surgery is closely associated with postoperative complications. Hypotensive events are more likely during major open vascular surgery. We prospectively investigated whether our institutional algorithm of cardiocirculatory management, which included the Hypotension Prediction Index (HPI), a predictive model of hypotension of the Hemosphere™ platform (Edwards Lifescience, Irwin, CA, USA), was able to reduce the incidence and severity of intraoperative hypotension during open abdominal aortic aneurysm repair.

**Methods:**

A multi-center observational study was conducted at IRCCS-Humanitas Research Hospital (Milan) and AO Mauriziano Umberto I Hospital (Turin) between July 2022 and September 2023, enrolling patients undergoing elective open abdominal aortic aneurysm repair. A hemodynamic protocol based on the Acumen-HPI Hemosphere™ platform was employed, integrating advanced parameters (e.g., HPI, Ea-dyn, dP/dt) and tailored interventions to minimize intraoperative hypotension. The primary endpoint was cumulative intraoperative hypotension time < 10% of surgical time, with secondary endpoints including incidence of hypotensive events, time-weighted averages of MAP < 65 mmHg (TWA65) and < 50 mmHg (TWA50), and postoperative complications.

**Results:**

We enrolled 53 patients submitted to open abdominal aortic repair. The primary endpoint (time in hypotension < 10%) was successfully reached: 5 [1–10] %. The targeted time-weighted average (< 0.40 mmHg) both for MAP < 65 mmHg (TWA65) and MAP < 50 mmHg (severe hypotension; TWA50) were reached: TWA65 = 0.26 [0.04–0.65] mmHg and TWA50 = 0.00 [0.00–0.01].

**Conclusions:**

Our hemodynamic management algorithm based on the HPI and other parameters of the Hemosphere™ platform was able to limit the incidence and severity of intraoperative hypotension during open abdominal aortic repair.

**Trial registration:**

NCT05478564.

**Supplementary Information:**

The online version contains supplementary material available at 10.1186/s44158-024-00222-x.

## Background

Intraoperative hypotension is common during noncardiac surgery [1, 2] and closely associated with serious complications, including acute kidney injury, myocardial infarction and increased mortality [3, 4].

Consequently, perioperative hemodynamic optimization, achieved by reducing the frequency, depth and duration of hypotension, has been associated with decreased organ injury [5, 6], and lower mortality and morbidity [7, 8].

The Hypotension Prediction Index (HPI, or simply *Index*) is a parameter on the HemoSphere™ advanced monitoring platform (Edwards Lifesciences, Irwin, CA, USA) that analyzes the arterial waveform, considering several features such as its time, amplitude, area, segment slopes, to predict hypotension (defined as mean arterial pressure (MAP) less than 65 mmHg for at least 1 min) [9, 10].

The *index* appears on the monitor as a numerical value ranging from 0 to 100, with higher numbers indicating a higher risk of hypotension several minutes before MAP actually decreases, potentially allowing clinicians to intervene and prevent hypotension [9, 11–13].

The system also provides advanced secondary hemodynamic information, such as cardiac output, dynamic arterial elastance (Ea-dyn), dP/dt (systolic slope), and stroke volume, which presumably helps the operator to treat the cause of the incoming hemodynamic failure [14, 15].

In fact, when the index begins to rise, secondary parameters can help understand the potential cause of the ongoing hemodynamic instability. Pulse pressure variation (PPV) and stroke volume variation (SVV) are markers of fluid responsiveness, Ea-dyn (which represents the PPV/SVV ratio) is related to pressure response to vasoconstrictor drug or fluid administration, and dP/dt is well correlated with the left ventricle elastance and reflects cardiac contractility [16–18].

The present study investigated the usefulness of the Hemosphere platform in limiting intraoperative hypotension in major open abdominal vascular surgery. We studied if the intraoperative HPI-guided hemodynamic management can potentially decrease hypotension severity and duration during open abdominal aortic surgery and consequently provide safer post-operative outcomes.

## Methods

A multi-centric observational study was conducted in IRCCS-Humanitas Research Hospital (Milan, Italy) and AO Mauriziano Umberto I Hospital (Turin, Italy).

All patients undergoing elective open repair of abdominal aorta aneurysm were enrolled between July 2022 and September 2023. Exclusion criteria were emergent/urgent procedure, age < 18 years, and pregnancy. Furthermore, we excluded those cases with intraoperative massive bleeding (> 3000 ml) because in such a situation, the cause of hemodynamic impairment is known, sudden occurring and then not predictable.

The aim of this study was to investigate whether a hemodynamic protocol based on the Acumen-HPI Hemosphere™ monitoring platform might be helpful to limit the incidence of intraoperative hypotension in terms of severity and duration in the context of open aortic surgery.

The study received the approval of the independent local Ethical Committee (Aut. n. 3236–19/07/2022) and was registered in ClinicalTrials.gov (NCT05478564).

For this observational study, we followed the STROBE (The Strengthening the Reporting of Observational Studies in Epidemiology) statement recommendations [19].

### Anesthesia and perioperative management protocol

All patients received intraoperative monitoring with invasive blood pressure, electrocardiogram, heart rate, peripheral oximetry, end-tidal carbon dioxide, and hourly urinary output. An arterial line was placed before induction and connected to an Acumen IQ sensor (Edwards Lifesciences, Irvine, California, USA). Trans-esophageal echocardiography was performed in selected cases according to the clinical judgment of the attending physician.

All patients received general anesthesia. Fentanyl, midazolam, and propofol were used for general anesthesia induction; oxygen-air mixture and sevoflurane plus remifentanil for the general anesthesia maintenance; rocuronium for myorelaxation. Mechanical ventilation was set as follows: Tidal volume 7–8 ml/kg; positive end-expiratory pressure 5 cmH_2_O; respiratory rate and minute ventilation were adjusted to maintain EtCO_2_ 30–35 mmHg and pCO_2_ 34–38 mmHg; FiO_2_ was set at 40–60%.

Maintenance fluid administration consisted of balanced crystalloids 4–5 ml/kg/h. Intraoperative blood management aimed to keep a level of hemoglobin > 7 g/dL. We used blood from a Red-Cell Saver (RCS) as first-line choice and Packed-Red Cells (PRC) from the blood bank if RCS was not enough to restore hemoglobin (Hb) levels above 7 g/dL.

Regarding post-operative pain control, a multimodal strategy was adopted. All the patients received a continuous infusion of ropivacaine 0.4% at 5 ml/h through a supra-fascial catheter inserted in the surgical wound at the end of the procedure. Pre-emptive local anesthesia was administered in the abdominal fascia with Ropivacaine 0.375% + Lidocaine 1% at the beginning of the operation. Multimodal post-operative pain control included oral pregabalin (300 mg) given once daily and I.V. paracetamol (1000 mg) given every 6 h. Rescue analgesia was provided by means of non-steroidal anti-inflammatory drugs.

All the patients were enrolled in our “fast-track” surgery protocol [20]: preoperatively, patients received 800 ml of clear drink containing 12.5% maltodextrin the preceding evening followed by an additional 400 ml up to 2 h before anesthesia. After surgery, standard cases were moved to the surgical ward. Intensive care unit (ICU) admission was limited to unplanned intraoperative complications as determined by the anesthetist in charge. Patients were encouraged to begin oral intake early, with a cup of tea allowed 4 h after anesthesia. Early mobilization was also encouraged, with patients sitting in an armchair 4–6 h after awakening and taking a brief walk 2–4 h later.

### Hemodynamic protocol

The hemodynamic management approach relied on the hemodynamic parameters offered by the HemoSphere platform featuring the Acumen IQ sensor. These include continuous Cardiac Index (CI), Stroke Volume Variation (SVV), and Acumen IQ-specific parameters such as maximum blood pressure rise (dP/dtmax), dynamic arterial elastance (Eadyn), and HPI.

**Fig. 1:**
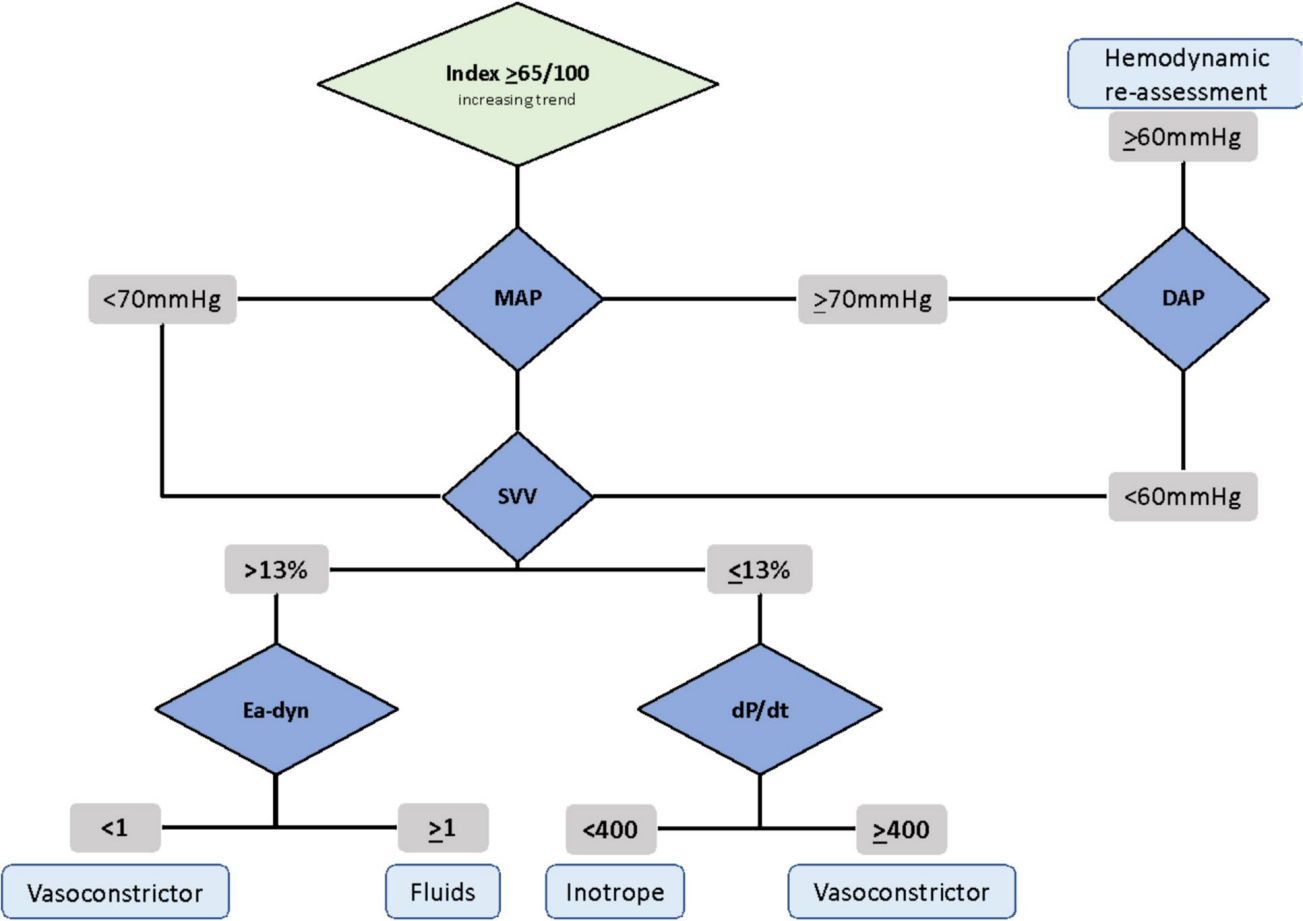
Hemodynamic management protocol

Our hemodynamic protocol was inspired by the “Predict H-Trial” flow-chart by Lorente-Olazabal et al. [21]. In our experience, we observed that HPI starts increasing when diastolic arterial pressure (DAP) drops < 60 mmHg although MAP is still within the normal range [22]. Therefore, we modified the original protocol by considering an HPI cut-off level of 65/100 and adding diastolic blood pressure (DAP) evaluation whenever the HPI alarm was triggered, but with MAP remaining > 65 mmHg [23]. The decision to adopt HPI 65/100 as the alarming level was since the most important factor to watch out is the rising trend of HPI. Consequently, when HPI is 85/100, it may be too late to act in limiting the hemodynamic impairment. Our hemodynamic management protocol is shown in Fig. 1.

In case of fluid dependence (SVV > 13%), Ea-dyn was checked as it represents a functional assessment of the vascular tone in a fluid-dependent setting, which guides the evaluation of fluid responsiveness. When Ea-dyn > 1, the arterial system should respond effectively to fluid administration with an increase in pulse pressure and systemic blood pressure. In this case, we administered a fluid challenge of Ringer Acetate of 4 ml/kg within 10–15 min or colloids (albumin 20% 100 ml) monitoring the fluid responsiveness within 3–5 min after the completion of the bolus.

Conversely, if Ea-dyn < 1, the pressor response to fluids was expected to be weak, thus the administration of a vasoconstrictor was the first treatment option. In this case, we administered norepinephrine (NE) 25 µg i.v. bolus, and, if necessary, after the third bolus, we started administering continuous NE targeting a decrease in the HPI at a value < 65/100. If an inotrope drug was needed, we tried ethylephrine (since it has β and α effects) 2 mg i.v. bolus. After the third bolus, we started dobutamine infusion with the aim of maintaining dP/dt > 400 mmHg/s [9–13].

The algorithm was adopted as a guide for hemodynamic management during the whole surgical period, including the post-clamping and post-unclamping phase of the operation. We are aware that the Acumen software cannot predict the cross-clamping and un-clamping, since these are two surgeon actions. But the aim of the algorithm was to make the patient arrive to those moments in the best possible cardiocirculatory conditions.

A poor adherence to the protocol was considered when > 20% of the times the operator ignored an action requested by the algorithm.

The threshold for blood transfusion was serum hemoglobin (Hb) < 7 g/dL (Hb < 10 g/dL for patients suffering from chronic coronary disease) after restitution of the blood from the red-cells saver.

Fluid balance included an estimation of *perspiratio insensibilis* of 5 ml/kg/h.

When fluid administration was requested, a bolus of balanced crystalloid solution (Ringer Acetate 4 ml/kg in 10 min) was administered. When the bolus resulted ineffective, a bolus of colloid solution (Albumine 20% 100 ml) was added.

### Outcomes

According to our clinical experience, before the implementation of an HPI-guided algorithm, the expected percentage of cumulative hypotension time was 16.1% of the surgical time (*unpublished data*). Based on this finding and assuming we can reduce the time spent in hypotension by adopting a hemodynamic protocol based on HPI, the primary endpoint of this study was the intraoperative cumulative time spent in hypotension lower than 10% of the surgical time.

The secondary endpoints were incidence of hypotensive events/patient (including the expected hypotensive event due to the aortic unclamping), the time-weighted average for MAP < 65 mmHg (TWA65) and postoperative complications. Moreover, we also investigated the TWA-MAP < 50 mmHg for severe hypotension (TWA50).

### Statistical analysis

Hypotension was defined as MAP < 65 mmHg for at least for 1 min. Severe hypotension was defined as MAP < 50 mmHg for at least for 1 min.

The intraoperative TWA65 was calculated as the area between the 65 mmHg threshold and the curve of the MAP measurements (AUC 65 mmHg), divided by the total continuous reading time [9]. We calculated the TWA both for hypotension (TWA65) and for severe hypotension (TWA50).

The two TWAs were calculated using the following formulas:

$$\mathrm{TWA}65=\lbrack(65-\mathrm{MAP})\times\mathrm{time}\;\mathrm{spent}\;\mathrm{in}\;\mathrm{hypotension}\rbrack/\mathrm{Duration}\;\mathrm{of}\;\mathrm{monitoring}$$

$$\mathrm{TWA}50=\lbrack(50-\mathrm{MAP})\times\mathrm{time}\;\mathrm{spent}\;\mathrm{in}\;\mathrm{hypotension}\rbrack/\mathrm{Duration}\;\mathrm{of}\;\mathrm{monitoring}$$

The advantage of using TWA-MAP instead of MAP lies in its ability to combine the severity and duration of hypotension, considering the overall duration of the surgery. Indeed, TWA is a marker of the severity, frequency and duration of hypotensive events and was used as a comprehensive assessment of the hypotension [13].

Other variables related to intraoperative hypotension included the number of intraoperative hypotension (IOH) episodes per patient and the total time of hypotension per case.

Data were recorded at four moments: pre-clamping, 5 min post-clamping, 5 min post-unclamping of the aorta, and after wound closure (end of surgery). Data were downloaded from the Hemosphere™ at the end of the procedure, and we collected considering the value of corresponding time of the phase of surgery. Subgroup analysis was performed to compare outcomes between patients who received vasopressors and those who did not as part of the hemodynamic management protocol. Furthermore, a secondary analysis explored the association between some risk factors (i.e. arterial hypertension) and TWA65.

### Sample size

The existing literature suggests that an overall hypotension time exceeding 10 min during surgery holds clinical relevance [24]. Due to the novelty of the Hemodynamic Prediction Index (HPI) parameter and the paucity of publications in the setting of vascular surgery, we referred to our clinical experience before the implementation of an HPI-guided algorithm. In our cohort of patients undergoing open repair of abdominal aortic aneurysms whose hemodynamics was managed according a FloTrac/EV1000 hemodynamic protocol, the percentage of cumulative time spent in hypotension was 16.1% (*unpublished data*). Based on this finding, we expected to find a mean of 10% of time spent in hypotension using the HPI protocol [25]. Thus, assuming the expected population standard deviation to be 5, and employing t-distribution to estimate sample size, the study would have required a sample size of 46 patients to estimate with 95% confidence and a precision of 1.5. Assuming a drop-out rate of 20%, we planned to enroll a total of 60 patients. With regard to secondary outcomes, in the same cohort of patients we found an average of 6.7 hypotensive events per patient and a TWA65 of 0.96 [0.25–1.39].

### Data analysis

A descriptive analysis was performed for the demographic and clinical characteristics of the study subjects. Continuous variables were reported as medians and interquartile ranges (IQRs). Categorical data were expressed as frequencies and percentages. Differences in categorical variables were assessed using either the χ^2^ test or Fisher’s exact test, depending on the appropriateness of the data. For comparisons of continuous variables between groups, the Wilcoxon signed-rank test or the Mann–Whitney *U* test were used for non-normally distributed data, while the Student’s t-test was employed for normally distributed data.

Analysis of variance (ANOVA) was employed for repeated measures for normally distributed data or the Friedman test for non-parametric data, enabling the examination of changes over time or across multiple experimental conditions. A two-sided significance level of *p* < 0.05 was considered for statistical significance.

Data analysis was conducted using R version 4.1.0 (The R Foundation). Missing data were not imputed.

## Results

**Fig. 2:**
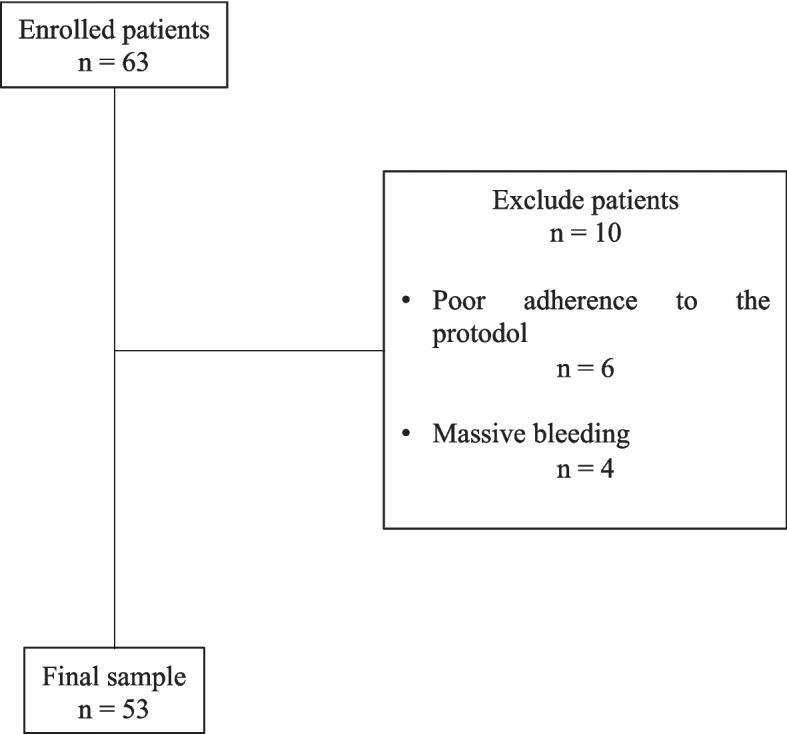
Flow-chart of the study

Between July 2022 and September 2023, we enrolled 63 patients submitted to elective abdominal aortic open aneurysmectomy. Ten cases were excluded because of poor adherence to the protocol (*n* = 6) and massive intraoperative bleeding (*n* = 4). The final sample included 53 subjects (Fig. 2).

The population characteristics are listed in Table 1.

**Table 1:** Study population

Age (year)	72 [68–77]
Gender (*n*, %)	
Male	35 (66)
Female	18 (34)
ASA physical status (*n*, %)	
2	20 (38)
3	30 (57)
4	3 (6)
BMI (kg/m^−2^)	25.3 [23.8–27.8]
Smokers (*n*, %)	38 (68)
Comorbidities (*n*,%)	
Hypertension	49 (88)
A/CKD	8 (14)
COPD	24 (43)
CAD	22 (39)
Arrhythmias	7 (13)
Medications (*n*, %)	
Beta-blockers	25 (45)
ACEi	23 (41)
ARB	16 (29)
CCB	12 (21)
Hemoglobin (mg/dL)	11.5 [10.5–13.0]
Pre-operative creatinine (mg/dL)	0.98 [0.86–1.23]
Aortic Clamping level	
Infrarenal	48 (91)
Suprarenal	5 (9)
NSQIP	
Any complications %	22.6 [19.6–25.1]
Serious complications %	19.6 [16.3–21.2]
Risk of death %	2.0 [0.9–3.2]
Cardiac complications %	3.5 [2.5–5.2]
Renal failure %	3.8 [2.4–5]

Data are presented as median (interquartile range) or *n* (%)

*ACEi* angiotensin-converting enzyme inhibitors, *ARB* angiotensin II receptor blocker, *ASA* American Society of Anesthesiologists, *A*/*CKD* acute/chronic kidney disease, *BMI* body mass index, *CCB* calcium-channel blocker, *NSQIP* National Surgical Quality Improvement Program

The median blood loss per patient resulted in 600 [450–1000] ml and the blood transfusion 275 [0–600] ml which involved 40 cases (75.5%). From these, six patients (11.3%) received red blood cells units from the blood-bank service, the other 36 cases (67.9%) were transfused only with blood from the red-cells saver. The final hemoglobin concentration resulted in 10.9 [9.6–11.8] g/dL. Fluid balance between patients who received norepinephrine and who did not, was significantly different (Table 2).

**Table 2:** Intraoperative fluids management

Crystalloids	
ml/kg/h	6.25 [5.2–8.1]
ml	2000 [1500–2300]
Colloids	
ml/kg/h	0.35 [0–0.77]
ml	100 [0–200]
Blood loss (ml)	600 [450–1000]
Diuresis (ml)	300 [200–450]
RBC transfusion^a^	
ml/kg/h	1 [0–1.95]
ml	275 [0–600]
End-of-surgery serum Hemoglobin	
g/dL	10.9 [9.6–11.8]
Fluid balance (ml)^b^	− 320 [− 585–351]

Data are presented as median (interquartile range)

*Abbreviations*: *RBC* Red blood cells

^a^Including blood from red-cells saver

^b^Including *perspiratio insensibilis* estimated as 5 ml/kg/h

The primary endpoint (time in hypotension < 10%) was successfully reached: 5 [1–10] %.

The secondary endpoints were the following: the number of hypotensive events per patient = 4 [1–6], TWA65 = 0.26 [0.04–0.65] mmHg, TWA50 = 0.00 [0.00–0.01], postoperative complication rate 21%.

The median aortic clamping time was 56 [47–70] min.

**Fig. 3:**
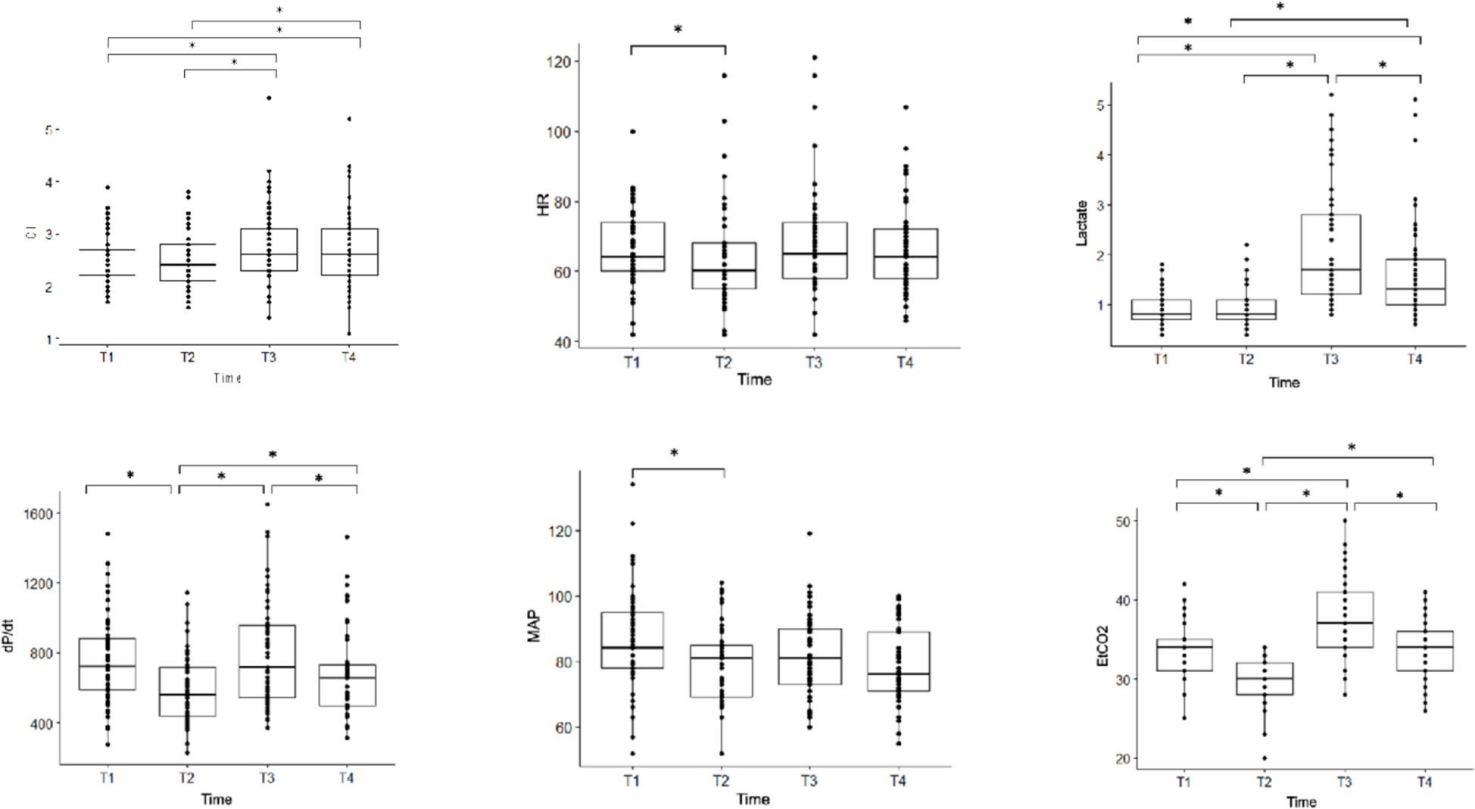
Hemodynamic parameters time-points differences. *p < 0.05. *CI* cardiac index (L/min/m^2^), *HR* heart rate (bpm), *Lactate* serum concentration of lactic acid (mmol/L), *dP*/*dt* pressure variation within the time-unit (mmHg/s), *MAP* mean arterial pressure (mmHg), *EtCO*_2_ end-tidal carbon dioxide (mmHg) *T1* basal, *T2* 5 min after aortic clamping, *T3* 5 min after aortic unclamping, *T4* end of surgery After aortic clamping (from T1 to T2), blood pressure, heart rate and dP/dt reduced significantly, while only dP/dt changed in the following phases

The median value of the hemodynamic parameters recorded during the four phases of the operation are listed in Table 3. Figure 3 shows the significant differences between the hemodynamic parameters.

**Table 3:** Intraoperative hemodynamic monitoring

	Pre-clamping	Post-clamping	Unclamping	End of surgery
MAP (mmHg)	84 [78–95]	81 [69–85]	81 [73–90]	76 [71–89]
HR (bpm)	64 [60–74]	60 [55–68]	65 [58–74]	64 [58–72]
EtCO_2_ (mmHg)	34 [31–35]	30 [28–32]	37 [34–41]	34 [31–36]
HPI	41 [19–70]	55 [33–96]	41 [22–75]	58 [25–91]
SVI (ml)	38 [33–42]	41 [33–45]	41 [34–46]	41 [35–47]
CI (l/min)	2.2 [2.0–2.7]	2.4 [2.1–2.8]	2.6 [2.3–3.1]	2.6 [2.2–3.1]
SVV (%)	10 [7–11]	13 [9–15]	8 [6–10]	7 [6–9]
dP/dt (mmHg/s)	720 [589–879]	559 [439–718]	716 [543–956]	655 [498–732]
Ea-dyn^a^	1.1 [0.9–1.2]	0.9 [0.7–1]	1 [0.9–1.2]	1.1 [0.9–1.2]
Serum lactate (mmol/L)	0.8 [0.7–1.1]	0.8 [0.7–1.1]	1.7 [1.2–2.8]	1.3 [1.0–1.9]
Serum hemoglobin (g/dL)	11.5 [10.5–13.0]	11.2 [10.2–12.4]	10.7 [9.6–12.1]	10.9 [9.6–11.8]

Differences statistically significant (*p* < 0.05) between the phases of the operation are showed in Fig. 2

Data are presented as median (interquartile range)

*CI* Cardiac Index, *Ea-dyn* Dynamic Arterial Elastance, *EtCO2* End-tidal Carbon Dioxide, *dP/dt* Rate of Change of Pressure, *HPI* Hypotension Prediction Index, *HR* Heart Rate, *MAP* Mean Arterial Pressur, *PPV* Pulse Pressure Variation, *SVI* Stroke Volume Index, *SVV* Stroke Volume Variation

^a^Ea-dyn (arterial dynamic elastance) is a dimensionless parameter since it is the PPV/SVV ratio

When we divided the sample in two sub-groups according to the administration of Norepinephrine, we did not observe any significant difference between them (Table 4).

**Table 4:** Vasopressors usage

	Whole population (*n* = 53)	Noradrenaline ( *n* = 37)	No noradrenaline (*n* = 16)	*p* value
TWA65 (mmHg)	0.26 [0.04–0.65]	0.22 [0.04–0.72]	0.31 [0.07–0.54]	0.469
TWA50 (mmHg)	0 [0–0.01]	0 [0–0.04]	0 [0–0]	0.107
Hypotension time (% surgery time)	5 [1–10]	4 [1, 2, 3, 4, 5, 6, 7, 8, 9]	7 [0–11]	0.605
Average MAP (mmHg)	80 [71–90]	80 [71–90]	81 [71–93]	0.979
Average HR (bpm)	64 [58–73]	62 [56–72]	66 [70–74]	0.680
Dobutamine (n,%)	7 (13.2)	6 (16.2)	1 (7.7)	0.660
Hypotensive episodes per patient	4 [1, 2, 3, 4, 5, 6]	3 [1, 2, 3, 4, 5, 6, 7]	4 [1, 2, 3, 4, 5, 6, 7, 8, 9]	0.459
Fluid balance (ml)^a^	0.26 [0.04–0.58]	− 0.720 [− 1297–590]	− 0.386 [− 1045–1069]	0.660

Data are presented as median (interquartile range)

*HR*, heart rate, *MAP* mean arterial pressure, *TWA* time-weighted average

^a^Including *perspiratio insensibilis* estimated as 5 ml/kg/h

After aortic clamping (from T1 to T2), blood pressure, heart rate, and dP/dt reduced significantly (*p* = 0.045, *p* = 0.023, *p* = 0.0002, respectively), while only dP/dt changed in the following phase (T2–T3 *p* = 0.00007; T3-T4 *p* = 0.146). According to the protocol, seven patients (13.2%) also needed inotrope support (Dobutamine 2–4 µg/kg/min) to correct dP/dt and maintain it in normal range throughout surgery (Fig. 3).

EtCO_2_, and serum lactate concentration changed coherently with the surgical phases. Hemoglobin variations showed a decreasing trend but were influenced by blood administration.

Eleven subjects were admitted to the ICU for postoperative observation and monitoring. In most cases they needed to stay one or two days overnight (Table 5).

**Table 5:** Outcomes

	Predicted incidence (%)	Actual incidence (%)	*p* value
Any complication	22.6	21	0.746
Cardiac complications	5	2	0.345
Renal complications	5	8	0.524
Death	3	0	0.203
Hospitalization			
ICU (n,%)		11(21)	
ICU length-of-stay (days)		1 [1, 2]	
Hospital length-of-stay (days)		4 [3, 4, 5]	
30-days mortality		0 (0)	

Data are presented as median (interquartile range) or *n* (%)

*ICU* intensive care unit

The predicted rate of postoperative complication by National Surgical Quality Improvement Program (NSQIP) score was 22.6 [19.6–25.1] % (Table 1). As listed in Table 4, the rate of complications in the sample was 21% as follows: two cardiovascular adverse events (atrial fibrillation), three respiratory (3 atelectasis without pneumonia), eight  acute kidney injury, and 3 others consisting of one gastrointestinal bleeding not needing surgery, two inferior limb ischemia requiring surgical treatment by Fogarty operation.

The difference between the predicted incidence of any postoperative complication by NSQIP (22.6 [19.6–25.1] %), and the actual postoperative adverse events (21%) rate was not statistically significant (*p* = 0.746 ).

Patients with postoperative complication showed an average value of TWA65 = 0.58 (IQR 0.34–1.17) while subjects with postoperative outcome without adverse events showed TWA65 = 0.19 (IQR 0.04–0.57) (*p* = 0.042).

We did not find any significant correlation between preoperative comorbidities/anti-hypertensive medications and TWA65 (Supplementary Table S5). Finally, the few episodes of severe hypotension (TWA50) did not affect the occurrence of postoperative complications (*p* = 0.415).

## Discussion

The main result of our study consisted of safe hemodynamic management during open abdominal aortic vascular repair, in terms of limiting the incidence and the severity of intraoperative hypotension.

The primary endpoint, defined as the intraoperative cumulative time spent in hypotension < 10% of the total surgical time, was successfully achieved. The secondary endpoints, which included the incidence of hypotensive events and TWA65, did not have a specific reference value for comparison. Nevertheless, these secondary outcomes were better than the unpublished data from our clinical experience prior to implementing the HPI-guided algorithm, which reported an average of 6.7 hypotensive events per patient and a TWA65 of 0.96 [0.25–1.39]. No recent data on postoperative complication rates were available for comparison, as these were not collected in our preliminary report.

In the highly specific context of aortic surgery, the implementation of a HPI into a comprehensive hemodynamic management algorithm led to a reduction in hypotension in terms of severity, duration and frequency of hypotensive events.

Aortic surgery remains a unique context because hemodynamic variations associated with clamping and unclamping make it virtually impossible to eliminate hypotensive episodes. Still, when unavoidable hypotensive events occurred, these was never severe (no MAP < 50 mmHg) nor frequent (average 3–4 episodes per patient). Indeed, in our sample, the median rate of time spent in hypotension was 5% of the total monitoring time. The time-weighted average (TWA65) for hypotension resulted 0.26 mmHg. Compared to our previous population treated with a hemodynamic algorithm based on FloTrac, the result is clinically significant (FloTrac TWA65 = 0.96 mmHg—data not published).

It is challenging to make a comparison with the literature because there is a lack of data on the population undergoing abdominal aortic surgery, and previous studies have grouped this surgical population with others, resulting in study populations that are too heterogeneous to allow for meaningful comparisons [26, 27]. In addition, we recruited a specific population of very fragile patients at high risk of intraoperative hypotension. This makes direct comparisons with other studies in different surgical contexts difficult, as the patient populations differ significantly in both size and comorbidities. As previously mentioned, compared to a benchmark from previous internal studies, we achieved a favorable outcome.

As our study does not include a formal comparison group, and we lack a reference TWA, we attempted to derive a point of reference from previously published studies. Specifically, the study by Wijnberge et al. focused on a general non-cardiac surgical population, which excluded vascular and liver surgeries, and can thus be considered at a lower risk of hemodynamic impairment compared to vascular surgery patients. Taking this into account, we hypothesize that a vascular surgery population would likely have a higher TWA than this general population. Wijnberge et al. reported a TWA65 of 0.44 mmHg in the control group (those not receiving HPI support). In contrast, our population showed a remarkable TWA65 of 0.26 mmHg despite the higher risk associated with vascular surgeries.

About the hemodynamic protocol adopted, we differ from what the manufacturing company recommends. Indeed, the HPI alarming threshold set by the manufacturer is 85, which is considered a cut off value beyond which the probability of hypotension in the subsequent minutes is very high. Based on clinical experience, we considered a different cut off HPI value as low as 65 to start the hemodynamic assessment and, eventually, treatment. This approach certainly led to maintenance of a stable hemodynamics, throughout all the phases of the operation. Still, some worries over the risk of overtreatment have been raised when using HPI [28–30]. To address these concerns, we analyzed separately patients who received norepinephrine and patients who did not. Indeed, we did not find any significant hemodynamic difference in terms of variations mean arterial pressure. We could infer that vasoactive drugs were used appropriately to ensure stable values of MAP, without any risk of “overshooting.”

In our experience, we noted that the *Index* starts to rise when the mean arterial pressure is still higher than 65 mmHg (in the range of the so-called “grey zone” of MAP between 65 and 75 mmHg) [22]. In the grey zone of MAP, overtreatment can be avoided or mitigated by careful analysis of the SSP and the diastolic arterial pressure. When HPI triggers an alarm with a MAP in the grey zone, evaluating altered diastolic pressure serves as a key factor in deciding whether to initiate treatment or not [22]. Indeed, it is the reduced vascular wall stretch, showing as decreased diastolic pressure, which triggers the compensatory mechanism to sustain arterial pressure when there is an injury affecting vascular tone, blood volume of heart function [25–27].

After the aortic clamping, MAP, heart rate (HR), and dP/dt (i.e., left ventricle myocardial contractility) reduced. General anesthetics impair cardiac function. A previous study showed that a consistent percentage of intraoperative hypotension (IOH) events are due to myocardial depression (40% of cases), bradycardia (56%), or to mixed causes, whereas vasodilation seems to be responsible for only 20% [25]. Moreover, the observed decrease of MAP and cardiac function in our population might have been expected since a few patients were affected by preexistent cardiac dysfunction and were under beta-blocking drugs.

In agreement with these findings and according to our algorithm, when dP/dt persistently reduced, in a patient who is not fluid-dependent, we started low dose of dobutamine (2–4 µg/kg/min) which was able to restore contractility (stroke volume increased) and to avoid hypotension. In addition, we also checked the cardiac function by trans-esophageal echocardiography as a supportive tool. In this sense, dP/dt was useful as a “screening tool” for in-depth assessment of the cardiac function.

Finally, apart from general anesthesia drugs and preexisting cardiac conditions, the aortic clamping itself could trigger a heart-pump dysfunction due to the rapid increase in the afterload after the occlusion of the aorta [26, 31, 32].

On the sideline of the above, despite NSQIP not being a specific score for vascular surgery, the predicted rates of major postoperative complications are consistent with our study. Even if this may signify that the outcome is affected by factors other than just the hemodynamics management, we also found that neither preoperative comorbidities, nor the anti-hypertensive therapy influenced the incidence of intraoperative hypotension, although we found that patients who had complications had a higher TWA. On the contrary, we may infer that the hemodynamic management was effective in limiting the expected hypotensive events due to the self-administered anti-hypertensive therapy which preclude the possibility of compensation by renin-angiotensin system, calcium-channels and hormone response due to the reduction of the stretch on the vessel walls when the cardiovascular injury is ongoing [25–27].

Our study has several limitations. First, while the use of the HPI provided valuable insights into hemodynamic trends, the findings of this study should be interpreted with caution, as the design does not allow for isolation of the HPI’s direct impact on postoperative complications. Instead, the observed outcomes are likely reflective of the overall hemodynamic management protocol, within which HPI was one component, rather than the index itself serving as the primary driver of the results.

Second, our results may be not valid for other type of surgery since aortic open repair is has some hemodynamic peculiarities due to clamping and unclamping of the aorta which are not common to other procedures. Furthermore, even in our centers some clinicians were reluctant to adopt a new proactive approach to avoid hypotension based on HPI algorithms in high-risk surgery, partly causing a drop out in the enrollment phase of the study. Fourth, due to the small sample size of the study population and the lack of a control group, our study was underpowered to detect clinical complications such as renal or cardiac ones. Further validation studies are necessary. Fifth, HPI relies on a high-quality arterial line waveform. If misfortunately the waveform is damped or fluctuates due to changes in patients position during surgery or other mechanical issues, the predictive ability of HPI might have been compromised. Finally, we did not record in how many patients the trans-esophageal ultrasound echocardiography was used and what impact it had on clinical decisions.

A final disclosure should be made about the dobutamine arm of our algorithm. In our study, the myocardial dysfunction observed in some patients during surgery was distinct from heart failure or cardiogenic shock. Indeed, this dysfunction did not lead to end-organ damage and was managed effectively with low-dose inotropes. Our approach began with boluses of alpha–beta agonists, such as ephedrine, to address transient contractility issues [33]. When contractile impairment persisted despite these measures, we introduced a low-dose inotropic infusion, typically dobutamine, which was sufficient to support cardiac function during critical phases, such as aortic clamping.

Importantly, the use of low-dose inotropes has not been associated with increased mortality in the literature [34]. On the contrary, evidence suggests that inotropes, when used judiciously or even prophylactically, may improve survival in specific surgical contexts. This is particularly relevant in high-risk patients undergoing procedures like open aortic repair, where goal-directed therapy has shown mortality benefits [35]. Furthermore, low-dose dobutamine, a common inotrope, is routinely used during stress echocardiography and is well-tolerated in that setting, further supporting its safety profile.

With this in mind, the inclusion of dP/dt in our hemodynamic monitoring protocol served as an early indicator of LV systolic dysfunction, providing valuable insight into contractile performance. In the setting of non-cardiac surgery, where transesophageal echocardiography may not always be readily available, dP/dt offers a practical screening tool to prompt further investigation and timely intervention. However, we acknowledge that dP/dt alone is not sufficient for a comprehensive assessment, hence its use should be approached with caution and always complemented by additional diagnostic modalities to ensure a thorough assessment of myocardial function.

## Conclusions

Since hypotension is associated with perioperative complications and adverse outcomes, high-risk patients undergoing open aortic aneurysm repair may benefit from hemodynamic management guided by HPI, which can reduce the occurrence of intraoperative hypotension. Indeed, in open abdominal aortic repair, HPI along with the other hemodynamic parameters proved to be helpful in managing the expected cardiocirculatory impairment due to characteristic of the patient (comorbidities and home therapy) and the surgical maneuvers (aortic clamping and unclamping) which deeply impact on the stability of the heart-vessels interaction.

## Supplementary Information

Supplementary Material 1.

## Data Availability

No datasets were generated or analysed during the current study.

## Abbreviations

ANOVA: Analysis of variance
AUC: Area under the curve
Ea-dyn: Dynamic arterial elastance
DAP: Diastolic arterial pressure
Hb: Hemoglobin
HPI: Hypotension Prediction Index
HR: Heart rate
IOH: Intraoperative hypotension
ICU: Intensive care unit
IQR: Interquartile range
MAP: Mean arterial pressure
NSQIP: National Surgical Quality Improvement Program
dP/dt: Rate of pressure change (dP) with time (dt) during isovolemic contraction of the cardiac ventricles
PPV: Pulse pressure variation
PRC: Packed-red blood cells
RCS: Red-cell saver
SVV: Stroke volume variation
TWA: Time-weighted average
