# Supplementary material for: Intraoperative hemodynamic management in abdominal aortic surgery guided by the Hypotension Prediction Index: the Hemas multicentric observational study
Source: J Anesth Analg Crit Care. 2025 Feb 13;5:7. doi: 10.1186/s44158-024-00222-x (PMC11823129; doi:10.1186/s44158-024-00222-x)
Supplement: Supplementary file 1 — Supplementary Material 1. [file 44158_2024_222_MOESM1_ESM.docx]

Additional files

Supplementary Table S1 Correlation between preoperative co-morbidities/anti-hypertensive medications and TWA65

|  | TWA65 | p value |
| --- | --- | --- |
| Smoke  Yes  No | 0.30 [0.02-0.75]  0.26 [0.07-0.65] | 0.758 |
| Arterial hypertension  Yes  No | 0.57 [0.31-0.91]  0.25 [0.04-0.64] | 0.223 |
| Obesity  Yes  No | 0.31 [0.04-0.67]  0.02 [0.0-0.63] | 0.199 |
| A/CKD  Yes  No | 0.26 [0.04-0.61]  0.45 [0.01-1.2] | 0.717 |
| COPD  Yes  No | 0.26 [0.04-0.55]  035 [0.0-1.0] | 0.374 |
| OSAS  Yes  No | 0.29 [0.04-0.65]  0.0 [0.0-0.0] | 0.178 |
| Cardiac ischemia  Yes  No | 0.34 [0.07-0.72]  0.21 [0.02-0.55] | 0.369 |
| Arrhythmias  Yes  No | 0.26 [0.04-0.67]  0.35 [0.0-0.68] | 0.673 |
| Chemo/radiotherapy  Yes  No | 0.31 [0.04-0.68]  0.02 [0.0-0.04] | 0.122 |
| Beta-blockers  Yes  No | 0.29 [0.04-0.77]  0.22 [0.04-0.67] | 0.775 |
| ACE inhibitors  Yes  No | 0.26 [0.05-0.67]  0.31[0.0-0.73] | 0.907 |
| Angiotensin receptor blockers  Yes  No | 0.26 [0.04-0.59]  0.35 [0.0-1.0] | 0.392 |
| Calcium channel blockers  Yes  No | 0.26 [0.035-0.60]  0.55 [0.05-0.83] | 0.991 |

Data are presented as median (interquartile range) or n (%).

Abbreviations: ACEi, Angiotensin-Converting Enzyme Inhibitors; A/CKD, acute/chronic kidney disease; COPD, Chronic obstructive pulmonary disease; OSAS, Obstructive sleep apnea syndrome; TWA, Time-Weighted Average.
